# Supplementary material for: Splice-Junction-Based Mapping of Alternative Isoforms in the Human Proteome
Source: Cell Rep. Author manuscript; Available in PMC 2020 Jan 15. (PMC6961840; doi:10.1016/j.celrep.2019.11.026)
Supplement: 2 [file NIHMS1546469-supplement-2.zip › DF1/PXD010154/Thyroid/Thyroid_26_TG_MELLASICWVSANIFEYQVDAQPLRPCELQR.pdf]

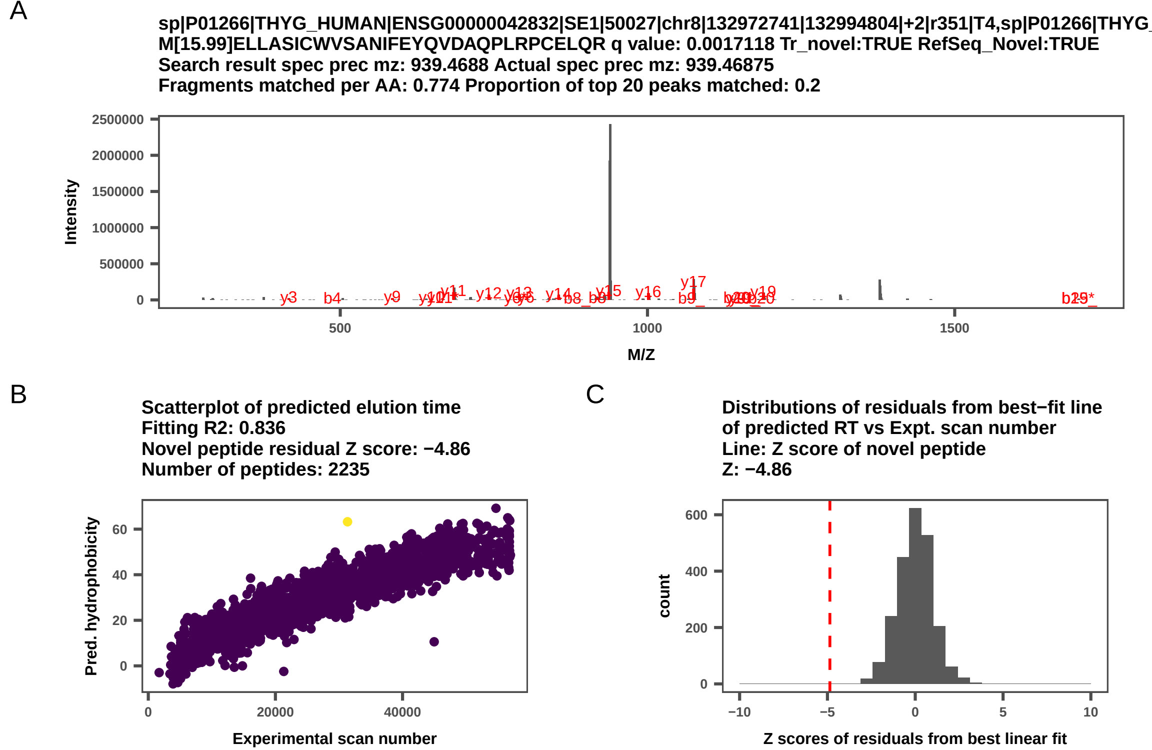
A
sp|P01266|THYG_HUMAN|ENSG00000042832|SE1|50027|chr8|132972741|132994804|+2|r351|T4,sp|P01266|THYG_
M[15.99]ELLASICWVSANIFEYQVDAQPLRPCELQR q value: 0.0017118 Tr_novel:TRUE RefSeq_Novel:TRUE
Search result spec prec mz: 939.4688 Actual spec prec mz: 939.46875
Fragments matched per AA: 0.774 Proportion of top 20 peaks matched: 0.2
Intensity
2500000
2000000
1500000
1000000
500000
0
y3
b4
y9
y11
y12
y14
y15
y16
y17
y19
500
1000
1500
M/Z
B
Scatterplot of predicted elution time
Fitting R2: 0.836
Novel peptide residual Z score: −4.86
Number of peptides: 2235
Pred. hydrophobicity
60
40
20
0
0
20000
40000
Experimental scan number
C
Distributions of residuals from best−fit line
of predicted RT vs Expt. scan number
Line: Z score of novel peptide
Z: −4.86
count
600
400
200
0
−10
−5
0
5
10
Z scores of residuals from best linear fit
